# Lipopolysaccharide Binding Protein and sCD14 are Not Produced as Acute Phase Proteins in Cardiac Surgery

**DOI:** 10.1155/2007/72356

**Published:** 2007-11-04

**Authors:** Manuela Kudlova, Pavel Kunes, Martina Kolackova, Vladimir Lonsky, Jiri Mandak, Ctirad Andrys, Karolina Jankovicova, Jan Krejsek

**Affiliations:** ^1^Institute of Clinical Immunology and Allergology, Charles University in Prague, School of Medicine and University Hospital in Hradec Králové, 500 05 Hradec Kralove , Czech Republic; ^2^Department of Cardiac Surgery, Charles University in Prague, School of Medicine and University Hospital in Hradec Králové, 500 05 Hradec Kralove , Czech Republic

## Abstract

*Objectives*. The changes in the serum levels of lipopolysaccharide binding protein (LBP) and sCD14 during cardiac surgery were followed in this study. *Design*. Thirty-four patients, 17 in each group, were randomly assigned to coronary artery bypass grafting surgery performed either with (“on-pump”) or without (“off-pump”) cardiopulmonary bypass. LBP and sCD14 were evaluated by ELISA. *Results*. The serum levels of LBP were gradually increased from the 1st postoperative day and reached their maximum on the 3rd postoperative day in both “on-pump” and “off-pump” patients (30.33±9.96 μg/mL; 37.99±16.58 μg/mL), respectively. There were no significant differences between “on-pump” and “off-pump” patients regarding LBP. The significantly increased levels of sCD14 from the 1st up to the 7th postoperative day in both “on-pump” and “off-pump” patients were found with no significant differences between these groups. No correlations between LBP and sCD14 and IL-6, CRP and long pentraxin PTX3 levels were found. *Conclusions*. The levels of LBP and sCD14 are elevated in cardiac surgical patients being similar in both groups. These molecules are not produced as acute phase proteins in these patients.

## 1. INTRODUCTION

Numerous events, potentialy generating inflammatory response, are induced during cardiac surgical operation on the open heart. Amongst them, the combination of surgical injury, mechanical manipulation with the heart, the contact of blood components with artificial surfaces of the cardiopulmonary bypass circuit, transient endotoxemia, and ischemia-reperfusion injury of the heart and lungs are relevant [1, 2].

The systemic inflammatory response syndrome (SIRS) viewed as basically useful has been conserved during evolution in order to provide support for the host to survive in an unfriendly environment, such as strenuous exercise of the “fight” or “flight” nature, multiple injuries or burns, infections or, more recently, major surgery. Whichever the underlying case, tight control of every step of the inflammatory reactions must be executed both on local and on systemic level where activities of the neuroimmune, endocrine, and circulatory systems overlap. If this control fails, morbidity and mortality increase dramatically [3].

The presence of bacterial lipopolysaccharide (LPS) or endotoxin in systemic circulation is sensed as a very strong danger pathogen associated molecular pattern (PAMP) by innate immunity. This identification could be followed by an exagerated and sometimes overwhelming systemic inflammatory reaction [4]. In cardiac surgical patients, transient endotoxemia is a manifestation of insufficient blood supply to the splanchnic vascular bed after a substantial amount of blood volume has been diverted from the patient's own vasculature into the tubing circuit of the heart-lung machine. Gut wall ischemia results in an increase of villous capillary permeability with ensuing translocation of lipopolysaccharide or even of the patient's own enteral flora into the systemic circulation.

The presence of LPS is identified by numerous phylogenetically highly conserved receptors of innate immunity which are called pattern recognition receptors (PRRs). PRRs are either humoral or membrane molecules which are sometimes shed into the body fluids. The crucial role in the identification of LPS is devoted to the humoral lipopolysaccharide binding protein (LBP) and two membrane receptors CD14 and toll-like receptor-4 (TLR-4, CD284), respectively. All these molecules are working in concert providing stimulatory signals to innate immunity cells such as monocyte-macrophages and granulocytes [5]. However, the former two molecules display a dual role in an inflammatory reaction. While present in plasma in low concentrations, CD14 being in the soluble form, early during inflammatory response, LBP and sCD14 are serving as a part of an early alarm system aimed at recognizing and binding of LPS and other danger signals, and thus enhancing the activation of the immune system. In the late phase, both sCD14 and LBP could play a role by preventing the lethal side effects of overwhelming inflammatory reaction induces by the presence of “danger.”

This study was aimed to follow the serum levels of LBP and sCD14 in cardiac surgical patients undergoing coronary artery bypass grafting (CABG) either with the use of cardiopulmonary bypass (on-pump) or without the use of cardiopulmonary bypass (off-pump). The levels of these markers immediately after surgery and up to seventh postoperative days were compared to preoperative level. Whereas LBP is recognized as a typical acute phase protein principally synthesized by hepatocytes, sCD14 molecules are either produced de novo as acute phase protein or are released into body fluids by shedding from cell surfaces. To ascertain the sources of sCD14, its level was correlated to the level of IL-6 which is the most potent stimulus for liver synthesis of acute phase proteins and to the levels of two pentraxins; C-reactive protein (CRP) and long pentraxin (PTX3), respectively.

## 2. PATIENTS AND METHODS

Forty patients (31 male, mean age 67.9±9 and 9 female, mean age 66.4±6.4, collective mean age 67.6±8.5 years) referred to first-time coronary artery bypass grafting (CABG) were enrolled in this study. Patients underwent either conventional myocardial revascularization with cardiopulmonary bypass and cardioplegic arrest of the heart (on-pump, n=2, 16 male, 4 females, mean age 69.4±7) or beating heart surgery (off-pump, n=2, 15 males, 5 females, mean age 65.9±9.7).

Patients in both groups were comparable in age, preoperative left ventricular ejection fraction (median 0.65 in on-pump, 0.65 in off-pump patients, resp.) and the number of performed coronary anastomoses (median 2.0 in on-pump, 2.0 in off-pump, resp.). All patients had been taking aspirin 100 mg in one daily dose, which was stopped for five days preceding the operation. Patients treated with anti-inflammatory agents, either steroids or NSAID, were excluded from the study, as were patients with serum creatinine ≥130 ≥mol/L or with hepatic disorders. No patients were known to suffer from concomitant malignancies. Patients with active infectious diseases are not admitted to elective CABG in our department. The study protocol was approved by the Ethics Committee of the University Hospital in Hradec Králové. All participants were informed in detail about the purpose of the study both orally and in writing. They were free to ask any questions. One person refused to participate for reasons he would not specify. All active subjects have given written informed consent.

Cardiopulmonary bypass, off-pump technique and anesthesiological management have been recently described in detail elsewhere [6].

## 3. BLOOD SAMPLING

Venous blood (central venous blood from arteria pulmonalis, peripheral venous blood from an antebrachial vein) was withdrawn in the operating room and on the first postoperative day in the ICU. Afterwards, only peripheral venous blood was taken due to the removal from the patients' vascular bed of all superfluous indwelling cannulas. Since there were practically no differences in results representative of blood samples originating from the respective sampling sites, for the sake of clarity only values obtained from the peripheral venous blood are indicated as results representative of the entire period of investigation. Samples were collected into tubes manufactured by Greiner, Germany.

In both on-pump and off-pump patients, blood was withdrawn at the following time points:

- introduction to anaesthesia, which in both groups represented the baseline or reference value for all parameters measured thereafter;
- after termination of the operation;
- the first postoperative day;
- the third postoperative day;
- the seventh postoperative day.

### 3.1. Blood sample analysis

Untreated blood samples were allowed to clot at room temperature. Serum samples were obtained after centrifugation (2000 g per 8 minutes), aliquoted and immediately freezed. Samples were thawed only once. Serum level of LBP was determined by ELISA kit, cat.number HK315, HyCult biotechnology b.v., The Netherlands. Serum level of sCD14 was evaluated by sCD14 EASIA kit, cat.number KAS0231, BioSource Europe S.A., Belgium. IL-6 was quantitatively measured by commercially available ELISA kit (BenderMed Systems) according manufacturer's instructions. Results were evaluated by spectrophotometry at 450 nm (Multiscan photometer) using Genesis software. CRP was assessed by immunonephelometry on IMAGE 800 (Beckman). PTX3 was detected using detection set (Alexis Biochemicals, Switzerland) cat.no. ALX-850-299-KI01 for sandwich ELISA application that provided capture monoclonal antibody to PTX3 (700 ng/mL), detection polyclonal antibody to PTX3 (25 ng/mL), and recombinant PTX3 (standard). Plates (96 wells, NuncmaxiSorb 446612) were read at 405 nm by an automatic reader (Multiscan photometer) and evaluated by Genesis software.

### 3.2. Statistics

Serum level changes within a time and differences between both groups of patients were compared by two-way analysis of variance for repeated measures and Fisher's post hoc test. Results are expressed as medians and quartiles. Relationships between concentrations of different cytokines were assessed using Pearson's correlation. Probability values of <.05 were considered statistically significant. Statistical analyses were performed with Statistica 6 software (StatSoft, USA).

## 4. RESULTS

### 4.1. Changes in the serum level of LBP

**Figure 1:**
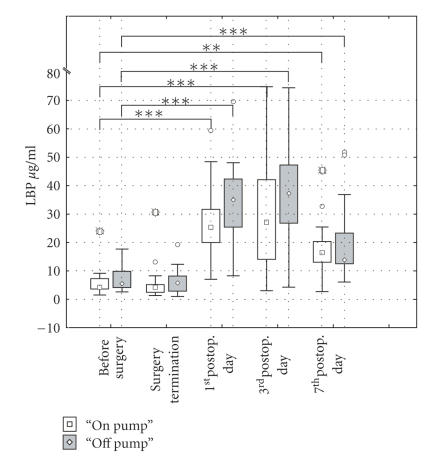
Comparison between serum levels of LBP in “*on-*” and “*off-pump*” patients F (1.32) = 1 .96; P < .l75.

The baseline preoperative levels of LBP to which LBP concentrations were statistically compared were nearly identical in both on-pump and off-pump patients (6.25±5.12 *μ*g/mL; 7.61±4.79 *μ*g/mL, resp.). The same situation was found after finishing surgery indicating that LBP levels are not influenced neither by surgery itself nor by cardiopulmonary bypass. The sharp statistically significant increase of LBP concentrations was found on the 1st postoperative day in both groups of patients. The maximal level of LBP was reached on the 3rd postoperative day in both on-pump and off-pump patients (30.33±9.96 *μ*g/mL; 37.99±16.58 *μ*g/mL, resp.). The serum levels of LBP declined therafter being still significantly higher on the 7th postoperative day in both on-pump and off-pump patients (18.11±9.96 *μ*g/mL; 20.15±13.79 *μ*g/mL, resp.). Suprisingly, the concentrations of LBP were slightly higher in off-pump patients comparing with on-pump patients. However, the statistical significance was not reached comparing on-pump and off-pump patients (P < .1715). Results are shown in Figure 1.

To test the hypothesis that LBP is serving as one of acute phase proteins, the correlation between serum levels of LBP and IL-6 which is the principal cytokine regulating acute phase proteins by hepatocytes, and the two members of prototypic pentraxin family acute phase proteins, C-reactive proteins, and long pentraxin 3, respectively, were tested. No correlations were found thus rejecting our hypothesis (data are not shown).

### 4.2. Changes in the serum level of sCD14

**Figure 2:**
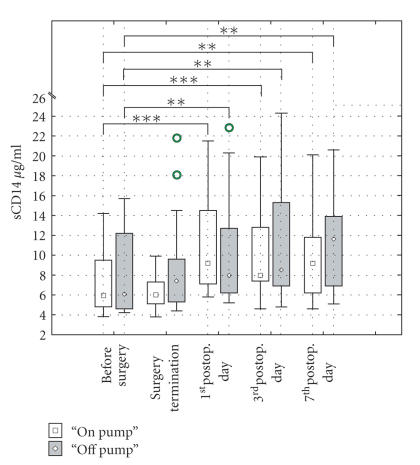
Comparison between serum levels of sCDI4 in “*on-*” and “*off-pump*” patients F (1.32) = 0.86; P < .4I7.

The baseline preoperative levels of sCD14 to which sCD14 concentrations were statistically compared were very similar in both on-pump and off-pump patients (7.29±3.32 *μ*g/mL; 8.30±4.26 *μ*g/mL, resp.). There was a slight, nonsignificant decrease of sCD14 level in on-pump patients after surgery (P=.170), whereas sCD14 concentration was nonsignificantly elevated in off-pump patients at that time. There were different trends in LBP concentration in on-pump and off-pump patients during postoperative period. The maximum of sCD14 concentration in on-pump patients was reached on the 1st postoperative day (11.30±4.93 *μ*g/mL; P=.0001) followed by a gradual decrease up to the 7th postoperative day (9.41±4.80 *μ*g/mL; P=.009) being still above preoperative baseline level. In contrast, there was a gradual increase of sCD14 serum level from the end of surgery up to the 7th postoperative day when the maximum was reached (11.05±4.57 *μ*g/mL; P=.002). In spite of these differences, no significant differencies between on-pump and off-patients were found (P=.417). Results are shown in Figure 2.

To test the hypothesis that sCD14 is serving as one of acute phase proteins, the correlation between serum levels of sCD14 and IL-6 which is the principal cytokine regulating acute phase proteins by hepatocytes, and two members of prototypic pentraxin family acute phase proteins, C-reactive proteins, and long pentraxin 3, respectively, were tested. No correlations were found thus rejecting our hypothesis (data are not shown).

## 5. DISCUSSION

Numerous danger patterns, both endogenous and exogenous origins, are generated in patients undergoing cardiac surgical operation. Sensing of these danger patterns via innate immunity pattern recognition receptors (PRRs) is followed by the development of inflammatory response. Numerous PRRs are now fully characterized. Amongst them LBP, CD14, MD-2, and TLR-4 are implicated as key factors in innate immunity cells activation by bacterial endotoxin [7].

LBP is a 50-kDa polypeptide mainly synthesized in hepatocytes and is released as a 60-kDa glycoprotein into blood stream after glycosylation. Other sources of LBP synthesis have been identified, such as epithelial cells of mucosa as well as the smooth muscle cells of lung arteries, and heart muscle cells. The amino acid sequence of LBP revealed substantial homology to bactericidal permeability increasing (BPI) protein, another LPS-binding protein originated in innate immunity.

LBP binds to the amphipathic lipid A moieties of LPS with high affinity and has been shown to facilite the process of LPS monomerization and subsequent presentation to other cellular and humoral binding sites. It catalyzes the transfer of LPS to a binding site of membrane-bound mCD14, which represents one part of cellular-LPS receptor. Adding LBP to a serum-free cell system enhances the LPS-mediated stimulation of CD14-positive cells 100- to 1000-fold. In addition, LBP transfers LPS to soluble sCD14 molecule [8].

We found significantly increased serum levels of LBP in our cardiac surgical patients from the first postoperative day up to the 7th postoperative day in comparison with preoperative level. The maximum, approximately 30 *μ*g/Ml, was reached on the 3rd postoperative day with subsequent decline in both off-pump and on-pump patients. It was suprizing to recognize that LBP level was even higher in off-pump patients, but no significant differences between on-pump and off-pump patients were found. We have no informations regarding LBP measurement in cardiac surgical patients to compare our results. The only one exception is the work by Fransen et al. [9] who followed LBP concentrations in on-pump patients. Unfortunately, their observation period was only up to 18 hours after declamping aorta. They found significantly increased LBP level at the 8th hour with subsequent increase at the 18th hour after start of reperfusion but maximum was not reached in their observation period.

Several in vivo and in vitro experiments demonstrated that LBP is a secretory class 1 acute phase protein whose gene is transcriptionaly activated by cytokine-inducible nuclear proteins. The transcriptional regulation is induced by IL-1 alone or synergistically by IL-1 and IL-6 leading to a maximum LBP concentration within 24–48 hours after stimulation. This response can be strongly enhanced by TNF-*α* and dexamethasone [10]. The dynamics of LBP concentrations in our cardiac surgical patients was resembling this kinetics but with prolongation up to the 7th postoperative day. To test the effect of proinflammatory cytokines on LBP synthesis, the level LBP was correlated with the level of IL-6 which is a principal cytokine regulating acute phase proteins synthesis in the liver. No such correlation was found (data are not shown). The possibility that LBP concentration in blood is influenced by exogenous corticosteroid is also unlikely. There was no significant difference between on-pump and off-pump patients despite the fact that former patient's group is exposed to methylprednisolone which is a standard component in CPB fluid used in our setting.

In humans LBP is constitutively in serum at concentrations of 5–15 *μ*g/mL. It is in a good concordance with its baseline level in our cardiac surgical patients. Its level is raised 10- to 50-fold during the acute phase reaction [11]. The similar findings were revealed by us in our patients. In contrast to C-reactive protein which level is peaked at the 3rd day in our patients and then rapidly declined, increased level of LBP is sustained up to the 7th postoperative day. It probably reflects the dual role of LBP in an inflammatory reaction. Whereas serving as a potent pattern recognition receptor at low concentrations early during inflammation to amplify the immune response rendering, for example, TNF-*α* production in macrophages, high concentrations inhibits danger-pattern-induced host cell activation [11]. It can be partly explained by the ability of LBP to transfer LPS to serum lipoproteins thus neutralizing the bioactivity of LPS. LPS has been shown to be physically associated with apoA- or apoB-containing lipoproteins and to transfer LPS into high- (HDL) and low-density lipoproteins (LDL) resulting in the clearance of LPS from the bloodstream. These capabilities were also reported for very-low-density lipoprotein (VLDL) and chylomicrons [12].

Up to now, few studies have been published evaluating the value of LBP as a diagnostic marker in patients with SIRS of noninfectious versus infectious origin and as potential prognostic marker predicting outcome [13]. These results can not be proven in our cardiac surgical patients because no cultivation-confirmed bacterial infections were found in this group. Information regarding LBP level during cardiac surgery are very sparse. It is reported by Vreugdenhil et al. [12] that plasma level of LBP is gradually increased from the 8th to 18th hour after declamping aorta but the maxim was not reached in their observation period. It is resembling our data but in our patients the maximum in LBP production was reached on the 3rd postoperative day. The role of LBP as acute phase proteins is unlikely in our cardiac surgical patients because no correlations with either IL-6, CRP, or PTX3 were found. It is extremly interesting that no significant differences in the serum level of LBP between on-pump and off-pump patients were found in our study. It is generally assumed that splanchnic hypoperfusion during extracorporeal circulation together with steady laminar blood flow that is generated by the heart-lung machine instead of the pulsative blood flow generated by each cardiac contraction, result in gut wall ischemia, subsequent increase of villous capillary permeability and transient endotoxemia. It is supposed that these changes are not so profound in off-pump patients. Lack of differences in LBP concentration between on-pump and off-pump patients could be interpreted in at least two ways. First, the intensity of the exposition to bacterial danger pattern is similar in both on-pump and off-pump patients. Second, LBP production is stimulated by another still unknown danger pattern which is identical in both on-pump and off-pump patients.

The second aim of our study was to follow the changes of soluble form of CD14 molecule (sCD14) during cardiac surgical operation. CD14 is a glycosylphosphatidyl-inositol-anchored protein constitutively expressed on the surface of various cells, including monocytes, macrophages, neutrophils, B-cells, dendritic cells, as well as several other cell types of nonhematopoietic origin. Aside of this membrane-bound state, CD14 is also found in a circulating soluble form [14]. CD14 molecule is the part of a receptor system of innate immunity cells to identify danger patterns of both exogenous and endogenous origin. This system is represented by the combined actions of the membrane-bound isoform of CD14 with the central transmembrane signaling unit of TLR-4 and the accessory protein MD-2 [15].

Two opposite functions have been described for sCD14. It can either reduce endotoxin-induced activities by competing with mCD14 for LPS binding or mediates the LPS-induced activation of non-CD14-expressing endothelial, epithelial, and smooth muscle cells. In addition, CD14 may function as a receptor for other microbial products, human heat shock proteins Hsp60, and other endogenous ligands such as ceramides, phospholipids, and modified lipoproteins [16].

We found significantly increased serum level of sCD14 from the 1st up to 7th postoperative days compared to the preoperative values in both on-pump and off-pump patients. Suprisingly, there are no significant differences between on-pump and off-pump patients. Evenmore, while reaching the maximum on the 1st postoperative day in on-pump patients, sCD14 level was gradually increasing in off-pump patients up to the 7th postoperative day (end of observation). It is not easy to discuss our findings. The level of sCD14 is not commonly assessed in cardiac surgical patients. It could be possible to extrapolate from patients undergoing major elective abdominal surgery as was reported by Hiki et al. [17]. They found slight decrease 6 hours after incision of skin, reaching the maximum on the 1st postoperative day thereafter decline to approximately baseline preoperative level on the 10th postoperative day. This pattern is resembling our off-pump patients with the exception that maximal concentration in our patients was slightly higher (11.3 *μ*g/mL versus 9.4 *μ*g/mL).

Several clinical studies have reported elevated serum levels of sCD14 in various inflammatory conditions such as Kawasaki disease [18]. Furthermore, correlation between sCD14 and severity of the trauma in polytraumatized patients have also been published [19]. Besides its function in LPS signaling, sCD14 might therefore play a role in inflammatory processes by controlling the immune system level of response. It has recently been demonstrated that sCD14 is a regulatory factor capable of modulating cellular and humoral immune responses by interacting directly with T and B cells [20]. Moreover, it has been suggested that sCD14 could be an acute phase protein, because apart from proteases-mediated shedding, sCD14 is also produced by hepatocytes, which represent the major source of acute phase proteins. Indeed, Bas et al. [14] in their clinical and experimental studies clearly showed that in patients suffering from rheumatoid arthritis, serum level of sCD14 did not correlate with the number of leukocytes, thus excluding an important source from leukocyte membrane-bound CD14, by protease-mediated shedding. In contrast, serum levels of sCD14 in these patients correlated with those of C-reactive protein, a classical acute phase protein, and IL-6, a cytokine known to regulate the synthesis of APP in the liver. We also sought for such correlations in our cardiac surgical patients. No statistically significant correlations between serum level of sCD14 and either IL-6 concentration or CRP and PTX3 levels were found in our study. It could be concluded from our results that sCD14 is not produced as one of acute phase proteins in cardiac surgery. The source of sCD14 in these patients remains enigmatic. Whether sCD14 in cardiac surgery originates mainly from leukocytes by protese-mediated shedding warrants further investigations.

In conclusion, we found significantly increased levels of both sCD14 and LBP in the early postoperative period in cardiac surgical patients. We found no significant differences between on-pump and off-pump patients in the serum levels of these parameters. Finally, both LBP and sCD14 molecules do not seem to act as acute phase proteins in cardiac surgical patients.

## ABBREVIATIONS

IL: Interleukin
LBP: Lipopolysaccharide binding protein
LPS: Lipopolysaccharide
PAMP: Pathogen associated molecular patterns
PRR: Pattern recognition receptors
PTX3: Long pentraxin 3
sCD14: Soluble form of CD14 molecule
SIRS: Systemic inflammatory response syndrome
TLR: Toll-like receptors
